# Systemic Treatment with Strontium Ranelate Accelerates the Filling of a Bone Defect and Improves the Material Level Properties of the Healing Bone

**DOI:** 10.1155/2014/549785

**Published:** 2014-08-28

**Authors:** Giovanna Zacchetti, Romain Dayer, René Rizzoli, Patrick Ammann

**Affiliations:** Division of Bone Disease, Department of Internal Medicine Specialties, Geneva University Hospitals and Faculty of Medicine, 1211 Geneva, Switzerland

## Abstract

Rapid bone defect filling with normal bone is a challenge in orthopaedics and dentistry. Strontium ranelate (SrRan) has been shown to in vitro decrease bone resorption and increase bone formation, and represents a potential agent with the capacity to accelerate bone defect filling. In this study, bone tibial defects of 2.5 mm in diameter were created in 6-month-old female rats orally fed SrRan (625 mg/kg/d; 5/7 days) or vehicle for 4, 8, or 12 weeks (10 rats per group per time point) from the time of surgery. Tibias were removed. Micro-architecture was determined by micro-computed tomography (*µ*CT) and material level properties by nanoindentation analysis. *µ*CT analysis showed that SrRan administration significantly improved microarchitecture of trabecular bone growing into the defect after 8 and 12 weeks of treatment compared to vehicle. SrRan treatment also accelerated the growth of cortical bone over the defect, but with different kinetics compared to trabecular bone, as the effects were already significant after 4 weeks. Nanoindentation analysis demonstrated that SrRan treatment significantly increased material level properties of both trabecular bone and cortical bone filling the defect compared to vehicle. SrRan accelerates the filling of bone defect by improving cortical and trabecular bone microarchitecture both quantitatively and qualitatively.

## 1. Introduction

Comminuted fractures, infection, dental extraction, bone metastasis, and orthopaedic surgery are among the main causes leading to a local loss of bone tissue [1, 2]. The age of the individual, hormonal status, nutrition, and presence of concomitant diseases may negatively affect bone tissue healing and the filling of the defect [3–5]. Bone defect healing occurs naturally after a phase of bleeding and inflammation and terminates with the formation of woven bone which is then remodelled by osteoclasts and replaced by lamellar bone by osteoblasts [6]. In cases of extensive bone loss requiring prosthetic fixation, as well as in fragile osteoporotic individuals, a treatment accelerating bone healing contributes to well-being and quality of life. Several approaches, such as the local or systemic administration of growth factors [7, 8] or mesenchymal stem cells [9–13], can represent advantageous alternatives to bone graft and the insertion of scaffolds of osteoinductive biomaterials.

The administration of anabolic or anticatabolic drugs commonly used to cure osteoporosis might promote bone defect healing by increasing bone formation or inhibiting bone resorption. Among these, the effect of the administration of antiresorptive agents to improve bone healing remains controversial. Bisphosphonates (BPs) do not interfere with the initial phase of bone healing, which is largely osteoclast-independent and leads to the filling of bone defect with woven bone. In some cases, BP administration appears to promote the formation of a larger callus [14] and suppression of the osteoclast activity delays the remodelling of woven bone and the formation of lamellar bone [15–17]. Importantly, treatment with BPs does not induce the regeneration of cortical bone after fracture [18]. Among the anabolic treatments, the systemic and local administration of parathyroid hormone (PTH) was shown to be efficacious for fracture and bone defect healing in rats and humans [19–21]. Depending on the anatomic localization of the bone lesion, the full repair of the cortical shell is crucial to restoring the load bearing capacity of bones. In osteoporotic nonfractured patients, PTH increases both trabecular bone volume and cortical thickness. However, it increases also cortical porosity, which may adversely affect bone strength [22].

By contrast, with bisphosphonates and PTH, strontium ranelate (SrRan) was shown to have a dual mechanism on bone formation activity and bone resorption mostly in vitro [23–27]. The effect of SrRan in vivo depends on the animal model. In rodents, long-term treatment with SrRan increased vertebral bone mass and had a positive effect on microarchitectural parameters, bone material level properties, and bone strength [28–30]. In monkeys, SrRan decreased histological markers of bone resorption, while preserving bone formation [31]. In ovariectomized rats, SrRan prevented bone loss [32] and had a beneficial effect on fracture healing, thus improving callus resistance to biomechanical torsional testing when compared to PTH [33–35].

The aim of this study was to assess whether the systemic administration of SrRan accelerates the healing of a bone defect created in rat proximal tibia compared to vehicle-treated controls.

## 2. Material and Methods

### 2.1. Animals and Diet

All experimental designs and procedures were approved by the Animal Ethics Committee of the University of Geneva, Faculty of Medicine. Sixty 6-month-old Sprague-Dawley female rats (Charles River Laboratories, L'Arbresle, France) were housed individually at 25°C with a 12:12-h light-dark cycle and strictly pair-fed a laboratory diet containing 15% casein, 0.8% phosphorus, 1% calcium, 70–80% carbohydrate, and 5% fat. Demineralized water was available* ad libitum*. Rats were then divided into six groups of 10 animals each. For a period of 4, 8, and 12 weeks after surgery, three groups (one for each time point) were treated with SrRan by gavage at a dose of 625 mg/kg/day, 5 days/week. This dose level leads to blood strontium concentration close to the level in human blood after a therapeutic dose of 2 g/day [32]. The three control groups received 0.5% carboxymethylcellulose aqueous solution by gavage 5 days a week for 4, 8, and 12 weeks with volumes corresponding to those administered in the SrRan-treated group.

### 2.2. Surgery

Animals were anesthetized with ketamine (100 mg/kg) and xylasine (10 mg/kg) administered as an intraperitoneal injection. Skin of both legs was shaved and cleaned with 70% ethanol. Under aseptic conditions, an anterior 10 mm incision was made to gain access to the proximal medial section of the tibia metaphysis. A standardized drill-hole defect (2.5 mm diameter, 2 mm depth, and approximately 10 mm^3^ total volume) was created in the proximal tibia secondary spongiosa of both legs using a dental burr under saline irrigation. The proximal limit of bone defect was delimited by a virtual line perpendicular to the long axis of the tibia and crossing the anterior edge of the growth plate centrally, which is curved both anteriorly and inferiorly in this central region. A second anatomical landmark was a virtual line from the inferior border of the tendinous insertion on the proximal anterior tibial crest to a medial tendinous insertion likely corresponding to the* pes anserinus* in humans. The bone defect was performed midway between these two tendinous insertions. Rotatory speed did not exceed 2000 rpm, and drilling was accompanied by profuse saline irrigation to avoid thermal bone necrosis. After creation of the bone defect, the skin was sutured using a 3–0 resorbable polyglactic suture (Vicryr; Ethicon; Spreitenbach, Switzerland). Blood was sampled before surgery and at the moment of sacrifice from the tip of the tail and the aorta, respectively. At the end of the experiments, all rats were sacrificed by an overdose of ketamine hydrochloride.

### 2.3. Microcomputerized Tomography (*μ*Ct)

Tibias were carefully excised immediately after death and frozen at −20°C in plastic bags. Bones were thawed slowly at 4°C and maintained at room temperature the night before *μ*Ct analysis. Each proximal tibia was scanned using *μ*Ct (*μ*Ct 40, Scanco Medical AG, Bassersdorf, Switzerland) as previously described [36, 37]. In summary, three-dimensional images of each tibia were acquired with a voxel size of 20 *μ*m in all spatial directions. No sample preparation was needed and tibia bones were secured in a cylindrical sample holder in NaCl solution. The resulting gray-scale images were segmented using a low-pass filter to remove noise and a fixed threshold to extract the mineralized bone phase. For detection of trabecular bone filling the defect, segmentation parameters were set to sigma: 0.8 voxels, support: 1, and threshold: 3.08 cm^−1^. The resolution was set to (500 projections with 1024 samples each), and a middle value slice thickness and increment to 21 *μ*m.

Trabecular bone was analysed by setting the volume of interest (VOI) as a circular band of 2.5 mm drawn on a slice-based method, starting from the first slice from the external bone surface containing no cortical bone and moving 30 slices dorsally, including avoidance of undrilled bone. Each slice was calculated directly from the binarized VOI. Total volume (TV) is the volume of the whole sample examined. Bone volume (BV) was calculated using tetrahedrons corresponding to the enclosed volume of the triangulated surface. Mean trabecular thickness (Tb.Th) was determined from the local thickness at each voxel representing bone [38]. Trabecular number (Tb.N) was calculated by taking the inverse of the mean distance between the middle axis of the structure and trabecular separation (Tb.Sp) by applying the technique used for the direct thickness calculation to the non-bone parts of the 3D image. Connectivity density based on Euler number (Conn. D) and the structure model index (SMI) were calculated.

**Figure 1:**
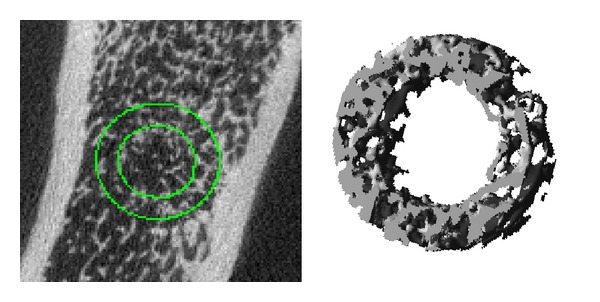
Micro-CT 3D reconstruction of a circular band 0.45 mm wide selected on the peripheral portion of a bone defect in adult female rat tibiae.

BV/TV, Tb.Th, Tb.N, Tb.Sp, and SMI were also analysed within a subregion of trabecular bone bordering the defect enclosed in a circular stripe of 0.45 mm and excluding the central cavity (Figure 1). For this analysis, the same parameters of segmentation as above were applied; the region of interest was selected within the volume of 30 slices previously analysed.

Scans were successively reformatted to the axial cuts to measure the thickness of cortical bone bridging the defect. The contours of cortical bone were semiautomatically drawned within 90 slices along the long axis of the tibia, exclusively including the cortices sealing the gap. For cortical bone, the segmentation parameters were set to sigma: 0.8 voxels, support: 1, and threshold: 3.85 cm^−1^.

### 2.4. Nanomechanical Testing

**Figure 2:**
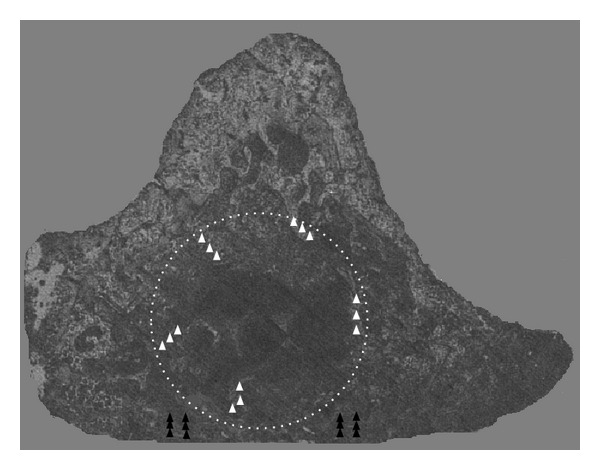
Schematic localization of indents on a representative section of proximal tibia metaphysis cut through the defect. The indents in the trabecular and cortical bone are represented by white and black triangles respectively.

Right tibias were embedded in polymethyl methacrylate (PMMA) (8.00590.2500 Merck, Hohenbrunn, Germany) and blocks were then transversally cut in two pieces at the level of the bone defect using a diamond wire saw (Well Mod 3242-3, Well Diamond Wire Saws SA, Le Locle, Switzerland). The face of the transverse cuts was polished and finished with 0.25 *μ*m diamond solution. After these preparation steps, specimens were frozen at −20°C. The night before the nanomechanical test, specimens were slowly thawed at 4°C, maintained at room temperature, and immersed in saline solution during the whole analysis. Nanoindentation was performed using a nanohardness tester (NHT; CSM Instruments, Peseux, Switzerland). In this test, force-displacement of a pyramidal diamond indenter that was pushed onto the bone was recorded. The nanoindentation tests included five indents within the bone defect and five indents at the junction between old and new formed bone in cortical bone (Figure 2). All the indents were performed at distance of the junction of the PMMA and bone. Indents were made up to 900 nm maximum depth applying an approximate strain rate of 0.066 1/s for both loading and unloading. At maximum load, a 5-s holding period was applied, and the limit of the maximum allowable thermal drift was set to 0.1 nm/s.

### 2.5. Wavelength X-Ray Dispersive Spectroscopy (WDS)

WDS was performed to evaluate the surface distribution of Sr, Ca, and P in the bone of five representative samples from the SrRan-treated and vehicle group (12 weeks of treatment only). Semiquantitative analyses were performed in profiles selected in trabecular bone at the edge with the defect in two representative samples for each treatment group (vehicle and SrRan) using a JEOL 8200 X-ray spectrometer (JEOL 8200 electron microprobe, Ohio, USA). The JEOL 8200 electron microprobe has five wavelength dispersive crystal focusing spectrometers. The crystals used here were pentaerythritol (PET), thallium acid phthalate (TAP), and synthetic 45 Å multilayer W/Si (LDE1). The primary electron beam energy was operated at 15 KeV, the electron beam current was 10 nA, and the beam spot size was ~10 × 8 *μ*m^2^. The bone samples utilized were embedded in PMMA and cut transversally in the middle, across the defect. The surface was polished, finishing with 0.25 *μ*m diamond solution, and coated with carbon to render them conductors and to avoid surface charging.

### 2.6. Biochemical Assay

Plasma insulin-like growth factor I (IGF-I) was measured by an ELISA kit (Immunodiagnostic Systems, Thebarton, Australia), according to the manufacturer's instructions.

### 2.7. Statistical Analysis

All results were expressed as means ± SEM. For normally distributed data, significant differences were identified by analysis of variance (ANOVA) and Fisher's* post hoc* test. Alternatively, a Mann-Whitney *U* test was performed, and the level of significance was set to *P* < 0.05.

## 3. Results

### 3.1. Effect of SrRan on the Geometry of Cortical Bone Bordering the Defect

**Figure 3:**
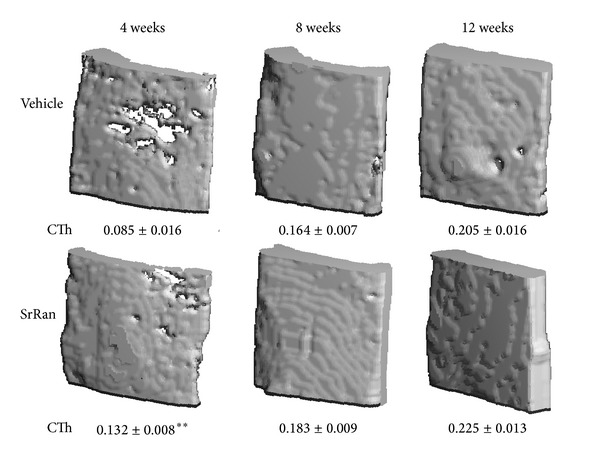
Micro-CT 3D reconstruction of cortical bone healing of a bone defect in proximal tibia metaphysis of adult female rats following 4, 8 or 12 weeks of vehicle or SrRan administration; values represent the cortical thickness (mm). Averages ± SEM, Anova **P < 0.001 versus time matched vehicle.

After 4 weeks of SrRan treatment, cortical bone thickness (+55%; *P* < 0.01) was higher than vehicle time-matched controls (Figure 3). *μ*CT analysis showed that the cortical bone healing area over the defect was almost completely restored in SrRan-treated rats at 4 weeks; at 8 and 12 weeks, the +12% and +10% higher cortical thicknesses were not significant. After 12 weeks, the cortical bone bridging the defect was fully repaired in both SrRan- and vehicle-treated rats.

### 3.2. Effect of SrRan on the Microarchitecture of Trabecular Bone Healing the Defect

**Figure 4:**
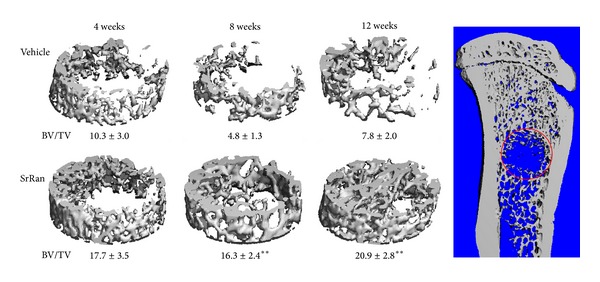
Micro-CT 3D reconstruction of trabecular bone healing of a bone defect in proximal tibia metaphysis of adult female rats following 4, 8 or 12 weeks of SrRan or vehicle administration; values represent the bone volume on total volume (%). Averages ± SEM, Anova **P < 0.01 versus time matched vehicle.

Most parameters of bone microarchitecture, such as BV/TV, Conn.D, and Tb.Th, were higher after 4 weeks of SrRan treatment (Figure 4 and Table 1) compared to the controls. A consistently higher trabecular BV/TV was observed at the early time point of 4 weeks and this difference became significant after 8 and 12 weeks of SrRan treatment (+72% (NS) and +240% [*P* < 0.01] and +168% [*P* < 0.01] at 4, 8, and 12 weeks, respectively, when compared to the time-matched controls). Tb.Th also followed the same trend as BV/TV at 4, 8, or 12 weeks of treatment (+13.6%, +31%, and +28%, resp.). Along with the higher Tb.Th, the SMI, corresponding to 0 and 3 for an ideal plate and rod structure, respectively, was lower by −19%, −35%, and −34% (4, 8, and 12 weeks, resp.) in SrRan-treated rats* versus* time-matched controls. A higher Conn.D was observed also after 4 weeks of SrRan treatment (+86%) and an even larger difference was detected after 8 and 12 weeks (245%, *P* < 0.01; 228%; *P* < 0.01, resp.) when compared to vehicle-treated rats. However, Tb.N was not increased at any time point following SrRan treatment. When analyzing trabecular bone by *μ*CT within a circular band of 0.45 mm at the periphery of the defect and omitting the central part of the cavity, the average BV/TV values measured in SrRan-treated rats were all above 20% (Figure 1 and Table 2). These values are higher than BV/TV measured in the secondary spongiosa of proximal tibiae in intact rats of the same age and strain (BV/TV = 15% [39]). By 12 weeks, higher BV/TV in this region was associated with significantly higher Tb.N. and Tb.Th and lower Tb.Sp and SMI* versus* controls.

### 3.3. Effect of SrRan on Material Level Properties of Cortical Bone Spreading from the Defect Limit

By 4 weeks of SrRan treatment a higher elastic modulus, hardness, and working energy of cortical bone bridging the defect (+37%, *P* < 0.001; +43%, *P* < 0.01; and +30%, *P* < 0.001, resp.) were observed when compared to vehicle-treated rats (Table 3). Working energy was also higher after 8 weeks of SrRan treatment, whereas values by week 12 were close to those observed in time-matched vehicles. By weeks 8 and 12, elastic modulus and hardness of cortical bone were similarly higher both in SrRan- and vehicle-treated groups. Bone material level properties were higher in cortical bone as compared to trabecular bone. The bone tissue organization and mineralization as well as intensity of mechanical loading [40] could account for this difference between cortical and trabecular bone.

### 3.4. Effect of SrRan on Material Level Properties of Trabecular Bone Broadening from the Defect Limit

By 4 weeks, SrRan treatment was associated with higher elastic modulus, hardness, and working energy of trabecular bone expanding from the defect rim (+26%, *P* < 0.01; +26%, *P* < 0.05; and +23%, *P* < 0.05, resp.) when compared to the time-matched vehicle-treated rats (Table 4). Elastic modulus, hardness, and working energy were higher by weeks 8 and 12 in both SrRan and vehicle groups; working energy remained significantly higher by week 12 of SrRan treatment when compared to time-matched vehicles.

### 3.5. Wavelength X-Ray Dispersive Spectroscopy

**Figure 5:**
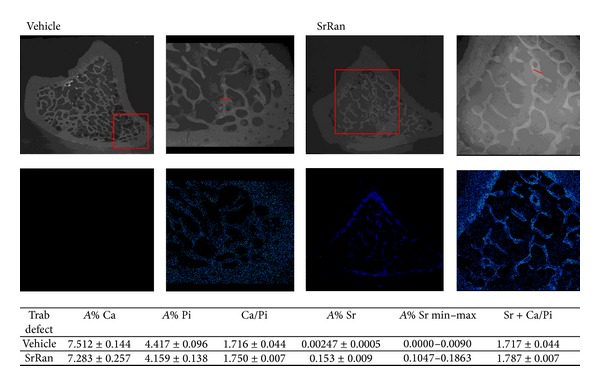
X-Ray spectroscopy of proximal tibia cross-section of two representative specimens following 12 weeks of vehicle or SrRan administration. Squares show strontium distribution in bone tissue in greater detail. Strontium deposits in both newly formed cortical and trabecular bone in SrRan treated rats, including the defect area. In the table, the atomic percent averages of Sr, Ca, and Pi were determined in profiles along trabecular units at the edge of the defect and are depicted by straight lines in the figure.

Elemental mapping of Sr in the bone defect of two representative samples at week 12 is presented in Figure 5. The mean atomic percentage of Sr, Ca, and P resulted from the evaluation of profiles selected in trabecular bone extending from the periphery of the defect. Sr was detected in trabecular bone healing the defect and all around in cortical bone, mainly in zones characterized by new and less mineralized bone. Sr was only detected in traces in time-matched vehicle rats. The mean percentages of Ca and P, as well as the ratio of Ca/P, were similar in SrRan and vehicle-treated rats.

### 3.6. Effect of SrRan Treatment on Insulin-Growth Factor (IGF-I) Level in Serum

Overall, the concentration of IGF-I lowered in serum harvested at the time of the euthanasia* versus* values measured before surgery (Table 5). Nevertheless, by weeks 4 and 8 the concentration of IGF-I was higher in SrRan-treated rats compared to time-matched vehicle animals, and by week 12, IGF-I levels were similar in both SrRan- and vehicle-treated groups.

## 4. Discussion

An accelerated repair of a bone defect represents a challenge to reconstruct bone integrity in individuals in whom bone loss is the consequence of traumatic events, surgery, or tooth extraction. SrRan has demonstrated some uncoupling between bone formation and bone resorption, as shown by a series of studies in vitro and in vivo [33–39, 41]. The aim of this study was to investigate whether the systemic administration of SrRan accelerates the healing of a bone defect drilled in rat secondary spongiosa of proximal tibiae. SrRan was orally administered at a dose of 625 mg/kg (5 days/week), and effects on bone defect repair were evaluated after 4, 8, and 12 weeks and subsequently compared with time-matched vehicle animals.

The *μ*Ct analysis showed that the mean cortical thickness was higher after 4 weeks of SrRan administration when compared to vehicle animals; in the latter group, the cortical shell bridging the defect still presented a few holes by 8 and 12 weeks, the cortices were thicker and formed a continuous shell in both SrRan- and vehicle-treated rats, completely sealing the defect. A former study showed that prolonged SrRan treatment increases bone diameter by inducing periosteal apposition and decreasing bone endocortical resorption [30]. The novelty of this study is in the evidence that SrRan accelerates the repair of damaged cortical bone. However, the mechanism by which SrRan influences the expansion of the cortical shell over the defect requires complementary investigations, that is, the histomorphometric quantification of parameters of bone formation/resorption.

The kinetics of trabecular bone healing of the defect seem to be delayed compared to those of cortical bone. Indeed, a significant increase in the parameters of cancellous bone microarchitecture was observed after 8 and 12 weeks of SrRan treatment* versus* time-matched vehicle animals, that is, 4 weeks later compared to the cortical bone. BV/TV and Tb.Th of cancellous bone filling the defect cavity were higher after SrRan treatment compared to vehicles. By contrast, the same region was still almost completely devoid of trabecular bone in rats treated with vehicle during the 12 weeks. This evidence clearly indicates that SrRan administration enhances the healing of a bone defect in rat tibiae. These observations are in agreement with other studies showing that SrRan treatment of rats carrying endosseous titanium cylinders in the proximal tibia increased the trabecular bone BV/TV at the bone-titanium interface, enhancing implant osseointegration [42].

The 3D reconstructions of *μ*Ct scans showed that trabecular bone heals from the periphery toward the centre of the defect. When excluding the central cavity, the analysis of trabecular bone located in a circular subregion of 0.45 mm at the periphery of the defect showed a significant increase in BV/TV in all groups of SrRan-treated rats. The mass and microarchitecture of cancellous bone after 12 weeks of SrRan administration were consistently improved when compared to values measured within the proximal tibia metaphysis of intact rats [39]. This suggests that SrRan administration improves the structure of bone healing the defect* versus* normal, undamaged bone. In addition, SrRan administration for a longer period of time might further improve the microarchitecture of bone extending through the central cavity from the periphery of the defect.

The SrRan-dependent increase in bone strength relies on the sum of positive effects on both microarchitecture and material properties as shown by a finite element analysis [43]. In the present study, the nanoindentation analysis showed a clear effect of SrRan on material level properties of both cortical and trabecular bone repairing the defect when compared to vehicle-treated rats. The increase in elastic modulus, hardness, and working energy was mainly observed after 4 weeks of SrRan administration* versus* time-matched vehicle animals, in both cortical and trabecular bone healing the defect. An effect of SrRan on working energy persisted in trabecular bone after 12-week treatment. In previous reports, it was shown that SrRan administration also improves bone strength by increasing the elastic modulus, hardness, and working energy in rat vertebrae [29, 30, 32]. A similar positive effect of SrRan on tissue quality was also observed in bone growing on implant surfaces [42]. To our knowledge, our findings provide evidence for the first time that systemic administration of SrRan improves the quality of healing bone in the context of a bone defect.

The incorporation of Sr in cancellous bone filling the defect was confirmed by WDS, while only traces were detectable in vehicle animals. In agreement with former reports, Sr was more concentrated in younger and less mineralized trabecular bone, including the cortical endosteal surface [44]. So far, the mechanism by which SrRan ameliorates tissue properties is unclear. Bone sustainability to mechanical deformation at nanometer scale is regulated by the interaction between collagen, noncollagenous protein, and mineral phase, which allows the load to be efficiently transferred across multiple structural levels through the slipping of matrix-fibrillar interfaces [45].

We hypothesised that the mechanism by which SrRan affects bone material properties may rely on a chemical effect on bone components (matrix and/or mineral phases), in addition to the biological effect on bone cells. This is supported by an ex vivo experiment, which showed the association between Sr content and the improvement of tissue properties in bone sections immerged in SrCl_2_ solution [46]. The unfolding of sacrificial bonds represents one possibility which allows bone tissue to withstand deformation. These interactions between mineral and organic phases act as a kind of “glue” opposing the separation of mineralized collagen fibrils [47, 48]. The adhesion of Sr to the hydrated layer may account for the improvement in tissue properties, as was proved by indentation performed under dry conditions in which no significant difference was observed between SrRan and vehicle-treated rats [30]. In addition, the hydration level of bone influences the interaction of the organic matrix with the mineral phase and dehydration is associated with a degradation of the plastic properties [49].

Previous findings have shown that Sr is mainly deposited in young bone, within the hydrated layer surrounding the hydroxyapatite crystals [50]. By contrast, the uptake of Sr ions in crystals in place of Ca^++^ ions is quite a rare event to induce an important change in bone material properties. This observation is of major importance as only young bone was formed in the defect in our model. We observed that only a borderline modification of the Ca/P ratio could be observed under SrRan treatment compared to the control group, and this observation is in agreement with the fact that Sr is essentially integrated into the hydrated layer and is rare in the hydroxyapatite crystal.

The mechanisms by which SrRan affects bone formation and trabecular bone remodelling in our model of bone defect were not specifically investigated in the present study. Multiple scenarios may be envisaged by referring to published in vitro and in vivo studies. The effect of SrRan on the production of hormones known to control cortical bone growth, such as IGF-I, might be incriminated. SrRan was demonstrated to increase plasma IGF-I in patients [51] and in rats [30]. In our study, SrRan administration was not associated with a significant change in plasma level of IGF-I and only a trend was observed. However, it cannot be excluded that SrRan might influence the local expression of IGF-I in bone. Interestingly, recent evidence has shown that the overexpression of IGF-I in osteoblasts of transgenic mice protected the bone microstructure from the negative effect of a low protein diet, despite the decrease in circulating levels of IGF-I [52]. The effect of SrRan on the fate of multipotent progenitors migrating through the site of injury at the time of blood vessel invasion during the early phases of bone defect repair must be taken into consideration. Indeed, it was shown that bone marrow stromal cells derived from ovariectomized rats treated with SrRan preferentially differentiate* versus* the osteoblastic lineage [53]. A more recent study showed that SrRan administration in mice lowers bone marrow adiposity and increases trabecular BV/TV in proximal tibia metaphysic [54]. Accordingly, in our model of tibial defect, SrRan may promote osteogenesis by downregulating genes driving the commitment of multipotent mesenchymal stem cells* versus* the adipocyte lineage and inducing genes involved in early phases of osteoblastogenesis. However another possible explanation may be that SrRan could positively influence defect vascularization by modulating the production of vascular endothelial growth factor (VEGF) from osteoblasts. In a recent study, it was shown in vitro that the release of strontium from strontium-doped calcium polyphosphate scaffolds was associated with the increase of VEGF mRNA and protein secretion from cultures of differentiating osteoblasts [55]. However, the association between SrRan administration and vasculogenesis in vivo has not been demonstrated yet.

## 5. Conclusions

In conclusion, our study demonstrates that the systemic administration of SrRan accelerates the healing of a bone defect created in rat proximal tibiae, with a significant effect on cortical thickness at 4 weeks and on trabecular microarchitecture at 8 and 12 weeks* versus* vehicle animals. Sr is integrated both in cortical and in trabecular bone healing the defect in SrRan-treated rats and improves the bone material level properties of the healing bone mainly after 4 weeks of treatment. These results open up new perspectives for the use of SrRan in clinical studies as a pharmacologic agent with a potential beneficial effect on bone defect repair.

## Figures and Tables

**Table 1:** Effect of 4, 8, or 12 weeks of SrRan or vehicle administration on micro-CT analysis of trabecular bone healing a bone defect in adult female rat tibiae.

Trabecular bone	Treatment	Time of treatment		
		4 weeks	8 weeks	12 weeks
Conn. D (1)	vehicle	40.56 ± 11.08	14.70 ± 4.49	22.86 ± 6.74
	SrRan	75.24 ± 15.15	50.73 ± 7.64∗	74.87 ± 11.62∗

TbN (1/mm)	vehicle	4.38 ± 0.33	3.75 ± 0.14	3.91 ± 0.16
	SrRan	4.60 ± 0.22	4.20 ± 0.10°	4.41 ± 0.17

TbTh (1/mm)	vehicle	0.066 ± 0.005	0.062 ± 0.003	0.064 ± 0.005
	SrRan	0.075 ± 0.005	0.081 ± 0.002^#^	0.082 ± 0.002∗

SMI (1)	vehicle	2.78 ± 0.27	3.30 ± 0.24	2.88 ± 0.30
	SrRan	2.25 ± 0.28	2.14 ± 0.13∗	1.91 ± 0.23°

Averages ± SEM; Mann-Whitney; °*P* < 0.05, *P < 0.01, ^#^ *P* < 0.001 versus time-matched vehicle.

**Table 2:** Effect of 4, 8, or 12 weeks of SrRan or vehicle administration on micro-CT analysis of a circular band of 0.45 mm wide selected on the peripheral portion of a bone defect in adult female rat tibiae.

Trabecular bone bordering defect	Treatment	Time of treatment		
		4 weeks	8 weeks	12 weeks
BV/TV (%)	vehicle	16.86 ± 4.61	14.16 ± 1.45	14.50 ± 1.86
	SrRan	26.57 ± 4.06°	22.84 ± 1.77°	27.86 ± 2.92∗

TbN (1/mm)	vehicle	5.00 ± 0.37	4.40 ± 0.11	4.46 ± 0.28
	SrRan	5.66 ± 0.40	4.99 ± 0.21	5.21 ± 0.18°

TbTh (mm)	vehicle	0.072 ± 0.007	0.082 ± 0.009	0.074 ± 0.003
	SrRan	0.083 ± 0.004	0.084 ± 0.002	0.084 ± 0.003°

TbSp (mm)	vehicle	0.217 ± 0.015	0.231 ± 0.006	0.248 ± 0.014
	SrRan	0.189 ± 0.014	0.216 ± 0.010	0.196 ± 0.008∗

SMI (1)	vehicle	2.487 ± 0.454	2.767 ± 0.137	2.505 ± 0.116
	SrRan	1.640 ± 0.368	1.706 ± 0.177°	1.544 ± 0.251°

Averages ± SEM; Mann-Whitney; °*P* < 0.05; *P < 0.01 versus time-matched vehicle.

**Table 3:** Effect of 4, 8, or 12 weeks of SrRan administration on material level properties of cortical bone healing a bone defect in adult female rat tibiae.

Cortical bone	Treatment	Time of treatment		
		4 weeks	8 weeks	12 weeks
Modulus (gPa)	Vehicle	8.31 ± 0.39	10.55 ± 0.37	14.87 ± 0.43
	SrRan	11.39 ± 0.60^#^	11.00 ± 0.71	14.40 ± 0.67

Hardness (mPa)	Vehicle	275.1 ± 17.8	291.3 ± 13.9	477.4 ± 23.0
	SrRan	394.5 ± 31.3∗	347.5 ± 29.3	482.8 ± 29.9

Working Energy (pJ)	Vehicle	1970.3 ± 96.4	1933.2 ± 68.4	3076.4 ± 102.6
	SrRan	2561.6 ± 113.1^#^	2269.5 ± 142.3°	3105.5 ± 123.3

Averages ± SEM; 2-way ANOVA; °*P* < 0.05; *P < 0.01; ^#^ *P* < 0.001 versus vehicle.

**Table 4:** Effect of 4, 8, or 12 weeks of SrRan administration on material level properties of trabecular bone healing a bone defect in adult female rat tibiae.

Trabecular bone	Treatment	Time of treatment		
		4 weeks	8 weeks	12 weeks
Modulus (gPa)	vehicle	9.17 ± 0.39	11.20 ± 0.53	13.50 ± 0.49
	SrRan	11.51 ± 0.58∗	11.66 ± 0.60	13.85 ± 0.43

Hardness (mPa)	vehicle	356.1 ± 24.2	507.31 ± 27.22	588.38 ± 19.48
	SrRan	449.6 ± 28.6°	534.51 ± 29.04	640-67 ± 20.06

Working Energy (pJ)	vehicle	2224.8 ± 135.9	2769.7 ± 139.4	3365.7 ± 104.2
	SrRan	2725.6 ± 145.8°	3006.3 ± 152.8	3758.2 ± 97.4∗

Averages ± SEM; 2 ways ANOVA; °*P* < 0.05; *P < 0.01 versus vehicle.

**Table 5:** Effect of 4, 8, or 12 weeks of SrRan or vehicle administration on IGF-I concentration in serum of adult female rats before surgery and after euthanasia.

Treatment	Surgery	4 weeks	8 weeks	12 weeks
vehicle	1181.1 ± 87.7	946.8 ± 50.8∗		
SrRan	1216.6 ± 77.9	1056.6 ± 49.1°		

vehicle	1034.1 ± 71.6		774.9 ± 82.0∗∗	
SrRan	1146.7 ± 47.9		966.2 ± 45.6°°	

vehicle	1196.7 ± 101.9			882.39 ± 79.37^†^
SrRan	1064.5 ± 79.2			886.39 ± 67.44

Averages (IGF-I; ng/mL) ± SEM.

4 wks: *P* < 0.05 compared to vehicle (∗) or SrRan group at surgery (°).

8 wks: *P* < 0.01 compared to vehicle (∗∗) or SrRan group at surgery (°°).

12 wks: *P* < 0.05 compared to vehicle group at surgery (^†^).
